# Redefining Breast Cancer Care by Harnessing Computational Drug Repositioning

**DOI:** 10.3390/medicina61091640

**Published:** 2025-09-10

**Authors:** Elena-Daniela Jurj, Daiana Colibășanu, Sabina-Oana Vasii, Liana Suciu, Cristina Adriana Dehelean, Lucreția Udrescu

**Affiliations:** 1 Center for Drug Data Analysis, Cheminformatics, and the Internet of Medical Things, “Victor Babeș” University of Medicine and Pharmacy Timișoara, 300041 Timișoara, Romania; 2 Departament II—Pharmaceutical Chemistry, “Victor Babeş” University of Medicine and Pharmacy Timișoara, 300041 Timișoara, Romania; 3 Department II—Pharmacology-Pharmacotherapy, “Victor Babeş” University of Medicine and Pharmacy Timişoara, 300041 Timişoara, Romania; suciu.liana@umft.ro; 4 Research Center for Pharmaco-Toxicological Evaluations, “Victor Babeş” University of Medicine and Pharmacy Timişoara, 300041 Timişoara, Romania; 5 Departament I—Toxicololgy, Drug Industry, Management and Legislation, “Victor Babeş” University of Medicine and Pharmacy Timișoara, 300041 Timișoara, Romania; 6 Departament I—Drug Analysis, “Victor Babeş” University of Medicine and Pharmacy Timișoara, 300041 Timișoara, Romania

**Keywords:** drug repositioning, breast cancer, computational drug repositioning, clinical validation

## Abstract

Breast cancer faces significant therapeutic challenges, particularly for triple-negative breast cancer (TNBC), due to limited targeted therapies and drug resistance. Drug repositioning leverages existing safety and pharmacokinetic data to expedite the identification of new indications with cost-effective benefits compared to de novo drug discovery. In this critical narrative review, we examine recent advances in computational repositioning strategies for breast cancer, focusing on network-based methods, computer-aided drug design, artificial intelligence and machine learning, transcriptomic signature matching, and multi-omics integration. We highlight key case studies that have progressed to preclinical validation or clinical evaluation. We assess comparative performance metrics, experimental validation outcomes, and real-world success rates. We also present critical methodological challenges, including data heterogeneity, bias in real-world data, and the need for study reproducibility. Our review emphasizes the importance of window-of-opportunity trials and the need for standardized data sharing and reproducible pipelines. These insights highlight the groundbreaking potential of in silico repositioning in addressing unmet needs in breast cancer therapy.

## 1. Introduction

Breast, ovarian, uterine, and prostate cancers are hormone-sensitive cancers that are responsible for a significant proportion of global cancer morbidity. Breast cancer is one of the most prevalent and life-threatening cancers worldwide, accounting for a significant portion of cancer-related deaths among women [1]. According to the World Health Organization 2024 report, 2.3 million women were diagnosed with breast cancer and there were 670,000 deaths in 2022 [2]. Recent advances in pharmacotherapy have significantly improved the outcomes of cancer patients and their quality of life [3]. However, resistance to conventional treatments remains a critical challenge [4,5,6]. In particular, triple-negative breast cancer (TNBC) has low or absent estrogen receptors (ERs), progesterone receptors (PRs), and human epidermal growth factor receptor 2 (HER2), so it lacks effective targeted therapies and is associated with a poor prognosis [7,8,9,10].

**Figure 1:**
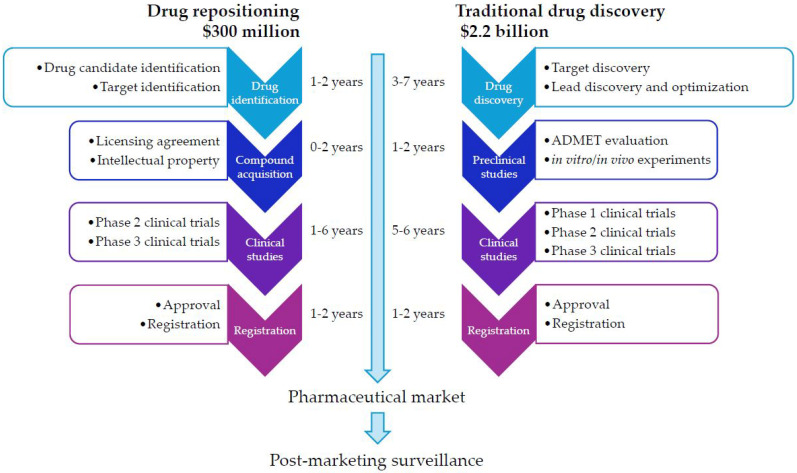
Comparative timelines, costs, and stages of traditional drug discovery versus drug repositioning.

The conventional drug discovery and development journey is a long, costly, and uncertain endeavor. It typically spans 10–17 years and has an average cost for 2024 of approximately USD 2.2 billion per new drug approved for clinical use [11,12,13] (Figure 1). Despite applying numerous effective strategies, 90% of clinical drug candidates still fail during various phases of clinical trials, prompting the examination of addressing critical elements related to drug development stages [12,14,15].

In this context, drug repositioning emerges as a powerful solution against the high failure rates of traditional drug discovery [16,17,18]. Drug repositioning capitalizes on the demonstrated safety profiles of existing drugs to uncover new uses for them; it also enables faster development timelines and lower costs than de novo drug development [19,20,21,22,23]. Breast cancer, driven by estrogen and progesterone receptor signaling in most cases [24,25], is an ideal clinical instance for drug repositioning due to its well-characterized biology and unmet needs, such as resistance to endocrine therapies.

Drug repositioning—or drug repurposing—has evolved beyond its original focus on approved medicines [21,26] toward a broader, World Health Organization-aligned usage that spans multiple development stages [16,27,28,29]. We outline these categories in Section 3.2.

In silico methods play a game-changer role in drug repositioning due to their great power to accelerate candidate identification by analyzing vast biological datasets. Computational drug-repositioning methods capitalize on artificial intelligence, machine learning, network pharmacology, and transcriptomics and have contributed significantly to identifying candidate drugs that entered clinical trials for testing the new indication. Computational methods can rapidly analyze large chemical, biological, and drug libraries, predict drug–target interactions, and identify molecular pathways associated with various pathologies [21,27,30,31,32,33,34,35].

This review brings forward the latest advances in computational drug repositioning for breast cancer, emphasizing key methods such as molecular docking, network-based and machine-learning-driven approaches, and omics-based drug discover. Furthermore, we illustrate notable case studies where computational predictions led to in vitro, in vivo, or clinical validation. Our review emphasizes the strengths and limitations of computational approaches in drug repositioning for breast cancer in hastening therapeutic development. Computational approaches followed by experimental validation hold immense promise in overcoming therapy resistance and improving clinical outcomes for breast cancer patients.

## 2. Brief Overview of Pathophysiology and Current Treatments of Breast Cancer

The heterogeneous nature of breast cancer biomarkers determines the four main groups of molecular subtypes—luminal A, luminal B, human epidermal growth factor receptor 2 (HER2)-enriched, and triple-negative breast cancer (TNBC). Luminal A tumors, which are strongly estrogen (ER)- and progesterone receptor (PR)-positive, typically have a favorable prognosis and are responsive to hormone therapy. Luminal B cancers also express ERs and PRs but may co-express HER2, resulting in more aggressive behavior. HER2-enriched tumors, typically with an ER-negative status, respond well to HER2-targeted therapies. TNBC, which lacks expression of ERs, PRs, and HER2, represents the most aggressive subtype and is frequently associated with a poor prognosis [36,37,38]. In clinical practice, ERs, PRs, and HER2 are essential predictive biomarkers for guiding endocrine and targeted therapies [39].

Adjuvant endocrine therapy is widely used in early-stage estrogen-receptor-positive breast cancer to mitigate the risk of recurrence [40]. The regimen varies based on menopausal status: tamoxifen, a selective estrogen receptor modulator (SERM), is prescribed for premenopausal and postmenopausal women, as well as for men with breast cancer. For postmenopausal women, tamoxifen is often combined with or followed by aromatase inhibitors such as letrozole or exemestane. The recommended minimum duration of endocrine therapy is five years, which provides a sustained reduction in recurrence risk lasting up to 15 years [39,40,41].

Advanced or metastatic hormone-receptor-positive breast cancer is typically treated with endocrine therapy that includes selective estrogen receptor modulators (SERMs) like tamoxifen and toremifene, anti-estrogens or selective estrogen receptor degraders (SERDs) such as fulvestrant and elacestrant, and aromatase inhibitors like anastrozole, letrozole, and exemestane, particularly in postmenopausal women [42]. Endocrine treatment is often used as first-line therapy, either as monotherapy or in combination with targeted agents [43]. Approved CDK4/6 inhibitors (abemaciclib, ribociclib, palbociclib) are added to endocrine therapy to enhance efficacy [44]. Additionally, immune checkpoint inhibitors (e.g., pembrolizumab, atezolizumab) are increasingly being combined with endocrine-based regimens in select cases, particularly in tumors with high PD-L1 expression, triple-negative subtypes, or specific genomic features, although their use in ER-positive disease remains an active area of investigation [45,46].

TNBC is associated with limited treatment options, frequent recurrence, and a poor prognosis. Given its lack of specific molecular targets, chemotherapy remains the primary treatment strategy, with higher rates of pathological complete response and improved outcomes compared to hormone-receptor-positive subtypes. Clinical guidelines recommend combination chemotherapic drugs, such as taxanes, anthracyclines, cyclophosphamide, cisplatin, and fluorouracil [8].

## 3. Methods

### 3.1. Review Methodology

We conducted a critical narrative review focused on computational drug repositioning for breast cancer. A literature search in PubMed/MEDLINE and Web of Science covered 2004, the year that Ashburn & Thor formalized drug repositioning [26], to April 2025. We used combinations of terms for drug repositioning/repurposing and breast cancer with method terms, such as network, artificial intelligence (AI), machine learning (ML), transcriptomic signature, docking, computer-aided drug design (CADD), multi-omics, and electronic health records (EHRs). We included peer-reviewed English-language articles reporting computational methods or resources for breast cancer repositioning, with or without in vitro/in vivo validation. We also included studies using real-world or clinical datasets when the analytic framework directly supported repositioning in breast cancer. We excluded articles limited to wet-lab testing of anti-breast-cancer activity without specifying the computational/systematic methodology that generated the candidate, as well as editorials, non-oncology repositioning without a transferable methodology, and studies lacking primary results. As a critical narrative review, we did not perform meta-analysis or formal risk-of-bias scoring; instead, we provide an interpretive synthesis and note limitations.

### 3.2. Terminology Note: Categories of Repositioning

Based on a drug’s starting development/regulatory status, we distinguish three categories of drug repositioning: (i) approved drugs (conventional repositioning) [21,26,29]; (ii) previously clinically tested drugs that failed for efficacy/safety or were withdrawn from the market [16,27,28,29]; and (iii) investigational (preclinical) compounds [29]. These categories differ in translational trajectory and regulatory expectations. Approved drugs leverage known safety profiles and chemistry–manufacturing–controls (CMC) packages and may progress via label expansion or investigator-initiated studies. Previously tested clinical candidates often retain partial safety/pharmacokinetic data but require renewed toxicology and intellectual property/regulatory assessment. Investigational compounds remain preclinical and therefore demand complete investigational new drug-enabling work before human studies.

## 4. Computational Methods for Drug Repositioning

Computational approaches to drug repositioning have undergone significant improvements with advances in bioinformatics, machine learning, and systems biology. The classification of these approaches can follow the primary data source or algorithmic framework used. This subsection explores key methodologies applied to hormone-dependent cancers, with a focus on breast cancer.

### 4.1. Network-Based Approaches

Complex network science emerged as a powerful computational tool in drug repositioning, as it provides a systems-level perspective to uncover novel therapeutic applications for existing drugs [30,47]. This approach represents biological systems as networks, entities as nodes (e.g., drugs, genes, proteins, or diseases), and interactions or relationships as edges (e.g., drug–drug, protein–protein, or drug–target). This methodology identifies hidden connections and predicts new drug properties beyond their original indications [19]. Network-based methods can map disease-specific pathways in a specific pathology and integrate multi-omics data to reveal key hubs or modules that drive that pathology [48]. For instance, network analysis can pinpoint repurposable drugs by assessing their proximity to disease-associated targets or identifying shared mechanisms across apparently unrelated conditions. Network centrality measures (e.g., degree, betweenness, closeness) and community detection algorithms increase the ability to prioritize candidates [49]. This holistic, data-driven technique accelerates repositioning and improves prediction accuracies; furthermore, these models are well-suited for integrating multi-omics data and can generate hypotheses beyond traditional single-target pharmacology.

The study by Chen et al. presents a computational approach to identifying breast and prostate cancer drug candidates by analyzing a human functional linkage network. Their network-based method integrates gene mutation, gene expression, and functional connectivity to detect drug–disease target interactions. They successfully identified FDA-approved drugs for breast and prostate cancer and many drugs in clinical trials, with higher performance than comparable methods, measured by ROC/AUC. In vitro tests on five candidates revealed higher inhibitory effects than Doxorubicin in MCF7 and SUM149 breast cancer cell lines [50].

F. Vitali et al. reported a network-based bioinformatics pipeline to identify multi-target drugs, particularly for triple-negative breast cancer (TNBC). They built a disease-specific protein–protein interaction network from multiple databases, employed graph-based algorithms to identify potential drug–target combinations, and applied a matrix tri-factorization data fusion technique. This pipeline rendered ranked lists of repositioning drug candidates (e.g., imatinib, L-aspartic acid, vemurafenib, hydroxyurea, azacitidine, flucytosine, and trametinib) and synergistic drug combinations (i.e., imatinib–vemurafenib, imatinib–flucytosine, and imatinib–vemurafenib–flucytosine), further confirmed by in vitro experiments [51].

NMF-DR (non-negative matrix-factorization-based drug repurposing) is a network-based model that consists of two steps: first, it preliminary processes data by building a method of analyzing and normalizing similarity matrices and building a weighted drug–disease heterogeneous network. The second step predicts drug–disease links using an improved NMF method. The authors validated their framework on breast cancer: the first five ranked repositioning candidates—i.e., leuprolide, estramustine, flutamide, bicalutamide, and mitoxantrone—are confirmed by the literature and clinical trials [52].

E. Jadamba and M. Shin [53] present a drug-repositioning framework integrating genomic and pharmaceutical knowledge. The authors built a pathway–drug network using disease-specific pathways and connectivity map (CMap) drug phenotype profiles, and then applied semi-supervised network propagation to predict repositioning candidates. They validated their method by testing 1309 drugs across four breast cancer datasets. Of the 17 drugs selected based on a weight criterion, 10 are established breast cancer drugs; the literature and published biological tests confirmed the potential benefits in breast cancers for 6 of the 7 remaining drugs (e.g., camptothecin, alsterpaullone, celastrol).

DRWBNCF is a neural collaborative filtering approach for drug repositioning. Using weighted bilinear graph convolution, it integrates drug–disease associations and drug–drug and disease–disease similarity networks. A multi-layer perceptron (MLP) optimized by α-balanced focal loss and graph regularization predicts novel drug–disease associations. The authors implemented their DRWBNCF model to predict new breast and small-cell lung cancer drugs. Valuable biomedical research sources and clinical trials validate as potential breast cancer drugs eight out of the top ten predicted medicines, such as metigestrona, estramustine, ethinylestradiol, estrone, norethisterone, daunorubicin, mitoxantrone, and leuprolide [54].

Another network-based drug-repositioning framework identified shared treatment options for breast and prostate cancers with DNA repair deficiencies, specifically targeting the homologous recombination (HR) pathway. The method integrated multi-cancer data and applied deep cross-cancer learning to uncover key biomarkers that guided the repositioning process. Through this approach, mitoxantrone and genistein emerged as potential therapeutics capable of reversing the gene expression signature associated with HR deficiency via a novel chemotherapeutic mechanism [55].

F. Firoozbakht et al. developed a network-based method associated with transcriptomic, genomic, and pathway data to find single or paired repositioning candidates for 10 breast cancer subtypes and TNBC. Their method recovers goserelin, an approved hormone therapy drug for breast cancer, in the top ten drugs for two distinct subtypes. This approach identifies ruxolitinib, which is already under investigation in several clinical trials, as a potential treatment for nine subtypes. In addition, the most promising drug pairs proposed for TNBC are bromocriptine combined with isradipine, emtricitabine, and etophylline clofibrate [56].

Recent work in graph-based AI has leveraged networks of drug–drug and drug–target interactions augmented with transcriptomic signatures to predict novel indications. One notable example is GraphRepur, which applies a graph neural network to integrate LINCS gene expression profiles with STITCH interaction graphs for drug repositioning [57]. We provide detailed descriptions of GraphRepur’s workflow and its clinical translation in Section 5.

### 4.2. Computer-Aided Drug Design Methods

Computer-aided drug design (CADD) employs pharmacophore modeling, virtual screening, and molecular docking for drug discovery and repositioning. While they share common principles, each technique has a distinct focus [58,59]. Pharmacophore modeling identifies key structural features for a drug to interact with a biological target using ligand-based or structure-based approaches [60]. Virtual screening is a broader category that screens large chemical databases to unveil potential drug candidates, incorporating ligand-based methods (i.e., similarity searches, quantitative structure–activity relationship (QSAR) models) and structure-based techniques (i.e., docking-based screening) [61]. Molecular docking is a structure-based method that predicts the binding mode of small molecules to a target protein at the atomic level, ranking their binding affinities by scoring functions [61]. It is commonly used to identify off-target interactions of existing drugs, a cornerstone of many repositioning efforts. These techniques are often included in a pipeline where pharmacophore modeling and virtual screening are preliminary steps followed by molecular docking and molecular dynamics simulations for further validation. These techniques offer mechanistic insights for rational drug design, particularly in drug repositioning, and are often used to prioritize compounds prior to in vitro validation.

Forkhead box M1 (FOXM1) is a key oncogenic transcription factor correlated with tumorigenesis and drug resistance in TNBC [62]. FOXM1 is overexpressed in TNBC and is associated with tumor aggressiveness, proliferation, poor patient outcomes, and reduced overall survival; this makes it an appealing molecular target for new therapeutic strategies.

A multi-step structure-based workflow was implemented to mine existing small-molecule libraries for FOXM1 binders. First, a pharmacophore model of FOXM1’s DNA-binding domain was built, and candidate compounds were docked into the predicted FOXM1 binding pocket. Top-ranked hits then underwent molecular dynamics simulations to evaluate binding stability and key interactions over time. From this pipeline, rabeprazole and pantoprazole emerged as top candidates, exhibiting predicted binding affinities in the low micromolar range. Subsequent in vitro assays in BT-20 and MCF-7 breast cancer cells confirmed, by in vitro tests, that rabeprazole and pantoprazole strongly inhibit FOXM1, reduce cell proliferation, and suppress downstream signaling in BT-20 and MCF-7 breast cancer cells. These results identify pantoprazole and rabeprazole as novel FOXM1 inhibitors, highlighting their potential for repurposing in FOXM1-driven breast cancers [63].

The structure-based drug-repositioning methodology developed by S. Majumder et al. [64] uses molecular docking and simulation techniques. This method exemplifies a computational repositioning strategy, integrating docking-based virtual screening and network-based pathway analysis: it evaluates the efficacy of simultaneously targeting PDPK1, ERK1/2, and mTOR in the MAPK pathway. Molecular docking employs AutoDock Vina to evaluate the binding affinities of known inhibitors and repurposable drugs against the three key proteins in the MAPK pathway. Molecular dynamics simulation validates the interactions. One thousand eight hundred and sixty-seven repositioned drugs were screened for potential multitarget activity to explore multitarget therapy by simultaneously targeting multiple nodes in a cancer-related signaling pathway to overcome drug resistance. This approach identified the zavegepant, an approved calcitonin-gene-related peptide (CGRP) receptor antagonist used for the acute treatment of migraine [65], as a promising candidate for overcoming drug resistance in breast cancer.

EGFR and HER2 are two key targets in cancer therapy, with lapatinib and gefitinib as the main inhibitors. In the study of [66], the authors conducted a ligand-based screening of the DrugBank database to identify FDA-approved drugs structurally similar to these two inhibitors. They analyzed the selected compounds using docking simulations and molecular dynamics to assess binding affinity and stability within inactive EGFR/HER2 conformations. Then, they identified the key residues contributing to binding stability for both HER2 and EGFR ligand complexes. They confirmed the in silico results with in vitro experiments by performing MTT assays on breast cancer cell lines MCF-7 and MDA-MB-231 to assess the antiproliferative effects of the repositioned compounds. Quinacrine and irinotecan exhibited higher antiproliferative activity than lapatinib and gefitinib in MCF-7 cells; quinacrine outperformed both standard drugs in MCF-7 and MDA-MB-231 cell lines.

Wnt pathway antagonists remain an unmet need in oncology. Koval et al. conducted a docking-based virtual screen (AutoDock Tools) of 1100 FDA-approved compounds against the Frizzled (FZD) receptor’s ligand-binding site. The top fourteen hits were assayed in TopFlash luciferase reporter assays, identifying four that blocked Wnt3a-driven transcription. Follow-up GTP-binding and β-catenin stabilization assays revealed clofazimine as a downstream inhibitor of canonical Wnt signaling. In vitro, clofazimine inhibited the Wnt pathway in HTB19 triple-negative breast cancer (TNBC) cell lines [67]. Based on their initial in vitro findings, the authors subsequently demonstrated that, in vivo, oral clofazimine dosing in two TNBC xenograft models resulted in robust tumor growth inhibition and on-target pathway suppression, with minimal toxicity. Moreover, clofazimine synergized with doxorubicin, demonstrating how CADD-driven repositioning can rapidly yield actionable leads in breast cancer [68].

### 4.3. Artificial Intelligence and Machine Learning Methods

Artificial intelligence (AI) and machine learning (ML) methods are crucial for speeding up drug repositioning due to rapid advancements in bioinformatics and the large amount of medical data available [69,70,71]. These vast datasets—from drug–drug and drug–target interactions and gene expression profiles to electronic health records (EHRs) and clinical outcomes—provide a rich foundation for discovering new therapeutic uses for existing drugs. AI/ML models can efficiently integrate and analyze these complex, high-dimensional datasets to identify hidden patterns between drugs, targets, and diseases and suggest opportunities to reposition candidates at a fraction of the time and cost compared to traditional methods. These approaches are highly scalable and can incorporate chemical, biological, and clinical features to prioritize candidates and generate mechanistic hypotheses for experimental follow-up. Their use complements, rather than replaces, experimental and clinical evaluation. In this context, the U.S. Food and Drug Administration (FDA), through the Center for Drug Evaluation and Research (CDER), noted growing AI use across the drug product lifecycle. To support the responsible implementation of AI in the pharmaceutical industry while balancing innovation and patient safety, the FDA is developing a risk-based framework. [72].

A. A. Jamali et al. [73] developed a machine-learning-based methodology for drug repositioning, precisely a weighted non-negative matrix factorization technique for predicting microRNA–drug interactions (MDIPA). The objective of utilizing neighborhood information on microRNAs and drugs, often applied in graph-based and collaborative filtering models, is to uncover novel drug–miRNA associations that could be significantly relevant for diseases such as breast cancer. MDIPA was compared to other methods and assessed with an independent dataset and cross-validation. Molecular docking confirmed this methodology for a breast cancer case study, predicting that gemcitabine binds to the oncogenic microRNAs mir-22 and mir-106a, while paclitaxel binds to mir-18A and mir-93.

A leading example in this category is GraphRepur, which leverages a graph neural network (GNN) on drug–drug networks augmented with transcriptomic features to achieve state-of-the-art predictive performance [57]. (Case study in Section 5).

Metformin has been highlighted by several EHR-mining and epidemiological ML studies as a candidate for breast cancer repositioning [74]. (Full case study in Section 5).

N.L. Tran et al.’s deep learning method [75] identifies the antiviral Z29077885 as a new candidate for cancer treatment targeting STK33. The methodology involved AI-based drug screening, in vitro validation using cell viability, Western blot, cell cycle, and apoptosis assays in MDA-MB-231 and A549 cancer cells, and in vivo confirmation in an A549 xenograft mouse model. Results showed that Z29077885 inhibits STK33 enzymatic activity, induces apoptosis via S-phase cell cycle arrest in lung and breast cancer cells, and exhibits significant anti-tumor efficacy in vivo. Z29077885 is an investigational (preclinical) compound rather than a conventional repositioning candidate, as it has no prior human/clinical trial exposure or approved indication. A more explicit framing as repositioning of investigational compounds would more accurately reflect the nature of Z29077885’s development trajectory, while still highlighting the strength of the AI-based strategy.

### 4.4. Transcriptomics and Gene-Signature-Based Methods

Transcriptomics—the study of RNA expression profiles—plays a crucial role in drug repositioning by identifying disease-specific gene expression patterns. Gene signatures—distinct sets of differentially expressed genes associated with a disease—match existing drugs that can reverse or modulate these expression patterns. Disease gene signatures are compared with drug-induced transcriptomic profiles from public databases (e.g., CMAP) to predict new indications for approved drugs. This approach accelerates drug discovery and drug repositioning based on molecular mechanisms.

Drug resistance in cancer treatment acutely requires the identification of effective therapies to improve patient outcomes. K. Yu et al. applied a rank-based pattern-matching algorithm to find compounds in CMAP that reverse drug resistance signatures. Their method predicted fulvestrant as a candidate for overcoming drug resistance; an in vitro test demonstrated that fulvestrant, combined with paclitaxel, enhanced the therapeutic response in a paclitaxel-resistant TNBC cell line (HCC-1937), overcoming resistance and surpassing the efficacy of paclitaxel alone [76].

The study reported by F. Khanjani et al. [77] used transcriptomic data from the Cancer Genome Atlas (TCGA) on HER2-positive breast cancer. The Linear Models for Microarray Data (limma) package identified differentially expressed genes (DEGs) between tumor and normal samples. These DEGs are input into the library of integrated network-based cellular signatures (LINCS) L1000CDS2 platform to predict candidates for repositioning. The tripartite network representing the relationships between repositioned drugs, target genes, and the genes associated with HER2-positive breast cancer revealed that vorinostat, mocetinostat, alvocidib, and curcumin are drugs with the highest potential for HER2-positive breast cancer therapy.

X. Zhou et al. [78] introduced an innovative drug-repositioning method: an ensemble of multiple drug-repositioning approaches called EMUDRA. First, they developed an expression-weighted cosine (EWCos) that minimizes the influence of lowly expressed genes on the drug–disease gene signature matching score. Then, they projected EMUDRA, which uses an ensemble framework to combine EWCos with three top-performing existing methods. EMUDRA significantly surpasses current state-of-the-art drug-repositioning techniques. This platform identified rifabutin as an agent for TNBC that was experimentally validated to inhibit the growth of MDA-MB-231 cells.

The study of [79] presents a pathway-based analytical approach using breast cancer transcriptomic data from TCGA and METABRIC. By leveraging high-throughput gene expression profiles from RNA-Seq and microarray platforms, the authors applied a robust probabilistic model to identify the most deregulated biological pathways at the subtype and patient level. Furthermore, the authors developed a database-based drug repurposing (DBDR) platform to support precision medicine strategies, with potential application at the patient level for personalized treatment; they proved its practicality in two individual breast cancer patients with known gene expression profiles, specific molecular subtypes, and main deregulated pathways: the analysis reveals the dysregulated pathways and their corresponding pathway deregulation scores (PDSs), identifies the related target genes, and highlights the drugs capable of restoring homeostasis within these pathways.

L. Li et al. [80] used a transcriptomics-based approach to reposition FDA-approved drugs to overcome acquired resistance to EGFR TKIs in lung cancer and to tamoxifen in breast cancer. To this end, the authors retrieved gene signatures associated with disease and resistance from the Gene Expression Omnibus (GEO). Then, they queried these signatures against the CMap database, which matches the transcriptional profiles of drug-treated cells to the disease profiles. The study analyzed five datasets for cancer vs. normal tissue and four datasets for therapy-sensitive vs. resistant states and identified 83 and 76 drug candidates, respectively; 12 FDA-approved drugs appeared in both groups, including aspirin, which was further validated in vitro and in vivo for its anti-cancer and anti-resistance effects. Aspirin reduced NF-kB activity in sensitive and resistant cells, effectively suppressing NF-kB signaling and counteracting targeted therapy-induced NF-kB activation.

Cuproptosis is copper-induced programmed cell death [81], with a crucial role in breast cancer progression [82,83]. In the study of [84], the authors developed a transcriptomic-based repositioning strategy to identify drugs capable of inducing cuproptosis. To this end, they extracted potential prognostic genes associated with cuproptosis in breast cancer from TCGA. Thereafter, using the prognosis genes and employing the CMap database, they found 22 candidate compounds with possible cuproptosis-inducing and anti-breast cancer activity. Fluphenazine, an antipsychotic drug, is a promising candidate further tested through wet-lab experiments. Experimental confirmation tests showed that fluphenazine significantly decreased cell viability in estrogen-receptor-positive (MCF-7) and triple-negative (MDA-MB-453, MDA-MB-231) breast cancer cell lines, suggesting fluphenazine as a potential anti-cancer therapy.

### 4.5. Multi-Omics Integration for Precision Drug Repositioning

Within multi-omics integration for precision drug repositioning, stratifying patients into biologically distinct subgroups is crucial for tailoring therapies to specific molecular profiles. For example, the study of [85] stratifies breast cancer (BC) patients into homogeneous subgroups based on shared clinical features and genotypic patterns (i.e., co-occurring gene expression regulation patterns); the authors contrasted these patterns with the rest of the population using gene–gene interaction and enrichment analysis. Then, they applied a graph-based drug-repositioning algorithm on a biomedical knowledge graph to rank drugs tailored to each subgroup. Analysis revealed that targeting antioxidants and ferroptosis may offer promising therapeutic strategies for TNBC. Most of the top-ranked repositioned drugs are kinase inhibitors—well-established cancer therapies; statins—drugs that can induce ferroptosis; and rifampicin, cerulenin, and lipoic acid—antioxidants with potential benefits in BC.

Table 1 summarizes various computational repositioning methods used in breast cancer to facilitate comparison between approaches. It highlights typical inputs, key considerations, such as pros and cons, and examples of validations.

## 5. Real-World Computational Successes, Limitations, and Clinical Uptake

Over the past decade, several repositioned drugs have progressed from computational prediction to preclinical or clinical evaluation in breast cancer, underscoring the translational power of in silico approaches. Table 2 highlights a few illustrative case studies to exemplify how computational drug-repositioning pipelines have successfully assigned and advanced existing drugs into clinical trials in breast cancer.

Cui et al. [57] developed GraphRepur, a graph neural network model for repositioning drugs against breast cancer. This method proposes selixenor, an approved drug for multiple myeloma, as a candidate for treating breast cancer. Other drugs that this method suggests as repositioning hints validated by the literature are mycophenolic acid, pitavastatin, dimethyl fumarate, ibrutinib, vismodegib, and sunitinib. A phase 2 clinical trial on selixenor in patients with advanced triple-negative breast cancer (TNBC) revealed that it was well tolerated with a good clinical benefit rate [86].

Xu et al. applied an NLP-driven ML framework to over 42165 electronic health records (EHRs) from the Mayo Clinic and Vanderbilt University; they identified a robust association between metformin use and reduced post-diagnosis mortality in breast cancer patients, even after adjusting for confounders such as smoking [74]. This observational signal, backed by numerous preclinical studies showing metformin’s anti-tumor effects across subtypes, motivated the MA.32 trial—a phase III, randomized, double-blind, placebo-controlled study conducted from August 2010 to March 2013 in Canada, Switzerland, the US, and the UK; the study involved 3649 non-diabetic, high-risk operable breast cancer patients (stratified by hormone receptor status, HER2 status, BMI, and chemotherapy) that received either 850 mg metformin or placebo twice daily for five years. The primary endpoint was invasive disease-free survival in hormone-receptor-positive patients, with key secondary endpoints including overall survival and distant relapse-free survival; metformin conferred no statistically significant benefit over placebo [87].

Although metformin’s journey, from the EHR-based hypothesis to the largest prospective trial of its kind, eloquently illustrates the power of computational repositioning to generate high-quality hints, it also highlights critical translational lessons. Retrospective associations, however compelling, may not predict therapeutic efficacy without careful patient selection (e.g., metabolic phenotype, tumor subtype) or combination strategies (e.g., with immunotherapy). Ultimately, MA.32 emphasizes that thorough, prospective validation is the ultimate measure of successful repositioning. Additionally, neutral or mixed trial outcomes provide valuable insights, as informative as positive results, for enhancing future in silico pipelines.

Danish health records showed a decrease in mortality among users of disulfiram for several types of cancer, including breast cancer. These real-world data findings, derived from retrospective EHR-style data, spurred interest in repositioning disulfiram [88]. For example, a phase II clinical trial (NCT03323346) is currently unfolding to evaluate the efficacy of disulfiram combined with copper in patients with metastatic breast cancer, aiming to establish clinical evidence for introducing this combination as a therapeutic option for patients who have exhausted conventional systemic or locoregional treatments [89].

Another example is the repositioning of propranolol for cancer, which was identified through large-scale observational studies. Perron et al. analyzed Canadian epidemiological records and reported a modest yet significant protective effect against prostate cancer in users of β-blockers [92]. This early signal highlighted the broader prospect of β-blockers in oncology. Barron et al. conducted a population-based analysis among Irish women and discovered that the use of propranolol in the year preceding breast cancer diagnosis was associated with a significantly reduced risk of advanced-stage disease compared to matched non-users; additionally, propranolol use corresponded with a dramatic reduction in breast-cancer-specific mortality [90]. These compelling real-world evidence studies established the foundation for subsequent phase II clinical trials, reinforcing propranolol’s repositioning journey from epidemiological signals to clinical evaluation [91,93].

## 6. Data-Driven and Methodological Gaps

Despite rapid advancements in computational drug repositioning for breast cancer, two major obstacle classes remain: shortcomings in the underlying data and limitations of the computational methods themselves.

Although data-centric approaches power modern repositioning pipelines, they also introduce unique challenges, such as data heterogeneity and bias. Real-world data (RWD) repositories—whether transcriptomic archives, electronic health records (EHRs), or patient registries—differ widely in format, depth, and quality. Gene expression profiles stem from either microarray or RNA sequencing platforms, each of which has distinct dynamic ranges and annotation patterns [94]. Furthermore, EHRs vary in coding conventions, missingness patterns, and their linkage to outcomes. These dissimilarities hinder direct aggregation and often lead to ad hoc assumptions, which can introduce systematic bias [95]. Cohort-based studies must also encounter confounders—such as comorbidities, socioeconomic status, and concurrent medications—that are unevenly captured in RWD, potentially yielding spurious repositioning signals [96].

Deep learning and graph-based algorithms excel at uncovering non-obvious patterns but often operate as “black boxes,” without mechanistic insight, limiting clinician trust and regulatory acceptance [97]. Explainable AI techniques—such as feature attribution and attention weights—are essential for justifying why a drug may have a new indication. Moreover, reproducibility requires open-source code and containerized environments, versioned data repositories to be re-run months or years later, and precise documentation of pre-processing steps and random seeds used in model training [70,98,99]. Despite larger datasets and more sophisticated AI, evidence remains largely retrospective or preclinical; rigorous external and prospective multi-site validation is needed [100]. Embedding computational predictions into adaptive or window-of-opportunity trial designs would significantly accelerate validation. Window-of-opportunity trials are short pre-treatment studies conducted between diagnosis and the start of standard therapy (e.g., before surgery). These trials involve administering a brief course of an investigational (often repurposed) drug and using paired pre- and post-treatment biopsies to assess pharmacodynamic and biomarker effects, without delaying standard care [101,102]. Although window-of-opportunity studies demand substantial resources, they provide an unbiased platform for assessing molecular activity, clinical efficacy, and mechanisms of action in vivo. Such designs yield robust, prospective evidence that can guide combination therapy hypotheses and biomarker development for personalized cancer treatment [103,104].

Standardized benchmarking suites, such as the Cancer Cell Line Encyclopedia (CCLE) and the Library of Integrated Network-Based Cellular Signatures (LINCS) datasets, open-source pipelines with containerized workflows, and increased sharing of prospective trial data will be essential to address these limitations.

## 7. Conclusions

Drug repositioning for breast cancer has matured into a diverse landscape of computational strategies that span network analysis, structure-based modeling, machine learning, transcriptomics, and multi-omics approaches. Each method contributes unique strengths. Network centrality metrics can uncover hitherto unrecognized drug–disease connections, while molecular docking and pharmacophore modeling yield mechanistic hypotheses. Artificial intelligence and machine learning (AI/ML) frameworks demonstrate state-of-the-art predictive power. Real-world data analyses highlight both the promise and risks of retrospective findings. However, translational gaps remain significant. Heterogeneity in gene expression platforms and electronic health records (EHRs) coding can introduce bias, and black-box AI models challenge clinical trust and regulatory acceptance. Moreover, moving a repositioning hypothesis from in vitro or animal validation into a confirmatory clinical trial requires considerable financial, logistical, and regulatory resources; this bottleneck helps to explain the slow pace of clinical follow-through for many computational candidates. Prospective validation, particularly through adaptive or window-of-opportunity trials, will be crucial to bridge in silico predictions with patient benefits. Additionally, scientific rigor and stakeholder confidence can be increased by standardizing data pipelines, sharing reproducible code, and addressing ethical considerations. Integrating explainable AI and real-time clinical feedback loops can further refine the prioritization of candidates.

By embedding computational pipelines within collaborative translational frameworks, this research area can accelerate the delivery of solid repositioning candidates to become safe, effective, and affordable breast cancer therapies.

## Figures and Tables

**Table 1:** Comparative summary of computational drug-repositioning methods for breast cancer.

Method Class	Input Data	Key Considerations	BC Validation Examples
Network-based	Drug–drug/target, protein–protein, disease–gene links; multi-omics overlays	*Pros:* systems view; integrates diverse sources; enables combo discovery and shared-mechanism mapping. *Cons:* interactome gaps; edge/source bias; sensitivity to priors; variable explainability.	Recovered BC candidates; in vitro > doxorubicin [50]; Hints (e.g., imatinib) and synergistic combinations (e.g., imatinib–vemurafenib) for TNBC confirmed in vitro [51]; Recovered confirmed drugs by the literature and clinical trials, e.g., leuprolide, [52], mitoxantrone [52,54,55], camptothecin [53], goserelin [56]; Drug pairs for TNBC (e.g., bromocriptine–isradipine) [56].
Structure-based, CADD	Protein structures; approved-drug libraries; pharmacophores; docking/MD	*Pros:* mechanistic hypotheses; interpretable interactions; rapid triage at scale. *Cons:* structure/pose/scoring bias; limited pathway context unless integrated.	Pantoprazole and rabeprazole validated in vitro as FOXM1 binders [63]; Zavegepant for drug resistance in BC [64]; Quinacrine > lapatinib and gefitinib in 2 BC cell lines [66]; Clofazimine inhibits canonical Wnt and suppresses TNBC growth in vitro/in vivo [67,68].
AI/ML	Chem-bio-clinical features; drug signatures; interaction graphs; EHRs	*Pros:* scalable integration; pattern discovery; fast prioritization, and hypothesis generation. *Cons:* data shift/bias; variable interpretability/reproducibility; requires careful external validation.	Identified novel drug–miRNA relevant for BC, e.g., gemcitabine and paclitaxel [73]; Z29077885 anti-BC in vitro and in vivo [75].
Transcriptomic signature matching	Disease DEGs (TCGA/METABRIC/GEO); drug profiles (CMAP/LINCS)	*Pros:* mechanism-aware; rapid hypothesis generation; subtype/patient tailoring. *Cons:* batch/platform effects; context mismatch; signature sensitivity.	Fulvestrant for paclitaxel-resistant TNBC [76]; Identified drugs for HER2+ BC (e.g., vorinostat) [77]; Rifabutin for TNBC in vitro [78]; DBDR patient-level therapy [79]; Aspirin counters NF-κB-mediated resistance in vitro/in vivo [80]; Fluphenazine cytotoxicity in ER+/TNBC in vitro [84].
Multi-omics integration	Genomic, transcriptomic, proteomic, clinical; knowledge graphs	*Pros:* aligns modality-specific signals; supports precision repositioning by subgroup. *Cons:* harmonization burden; small subgroup *n*; fused-feature interpretability.	Subgroup-tailored rankings: ferroptosis/antioxidants and kinase inhibitors for TNBC [85].

**Table 2:** A selection of case studies presenting the application of computational drug-repositioning workflows in identifying and advancing repositioned drugs for breast cancer clinical trials.

Case Study	Computational Approach	Data Source and Pipeline	Clinical Stage	Key Findings	Limitations and Conclusions
Selinexor	Graph neural network (GraphRepur) [57]	LINCS drug-exposure gene expression + STITCH drug–drug associations → Differential-expression analysis (breast cancer) → Extract drug signatures → Build drug–drug graph → Train GNN (GraphRepur) → Output repositioning scores	Phase I/II TNBC trial [86]	Well tolerated, partial responses in pre-treated TNBC	Requires larger trials; explainability of GNN predictions
Metformin	EHR-driven ML (NLP framework) [74]	>42,000 EHRs [74]; MA.32 phase III RCT (850 mg × 2 daily vs. placebo) [87]	Phase III trial (NCT01101438) [87]	Reduced mortality after a cancer diagnosis in both diabetic and non-diabetic patients [74]. No significant benefit compared with placebo [87]	Observational signals may not predict efficacy; need biomarker-guided stratification
Disulfiram/Copper	Real-world EHR analytics [88]	Danish health records—disulfiram [88]; epidemiological signal → phase II trial NCT03323346—disufiram + copper [89]	Ongoing phase II trial [89]	Retrospective reduction in cancer mortality; trial testing safety/efficacy in metastatic breast cancer	Need prospective validation; optimal dosing/combination unknown
Propranolol	Observational epidemiology [90]	Ireland registries: breast cancer patients using propranolol or atenolol [90]; propranolol use pre-diagnosis → reduced advanced-stage disease and mortality	Phase II clinical evaluation [91]	Protective association against metastatic progression and cancer-specific death [90]. Combining propranolol with neoadjuvant chemotherapy is feasible [91]	Confounding factors in observational data; mechanisms in breast cancer need study

## Abbreviations

The following abbreviations are used in this manuscript:

ADMET	Absorption, Distribution, Metabolism, Excretion, and Toxicity
AI	Artificial Intelligence
AUC	Area Under the Curve
BC	Breast Cancer
BMI	Body Mass Index
CADD	Computer-Aided Drug Design
CDER	Center for Drug Evaluation and Research
CDK	Cyclin-Dependent Kinase
CGRP	Calcitonin Gene-Related Peptide
CMap	Connectivity Map
EGFR	Epidermal Growth Factor Receptor
EHR	Electronic Health Record
ERs	Estrogen Receptors
ERK	Extracellular Signal-Regulated Kinase
FDA	The United States Food and Drug Administration
FOXM1	Forkhead Box M1
FZD	Frizzled Receptor
GNN	Graph Neural Network
HER2	Human Epidermal Growth Factor Receptor 2
HR	Homologous recombination
LINCS	The Library of Integrated Network-Based Cellular Signatures
MAPK	Mitogen-Activated Protein Kinase
MCF-7	Michigan Cancer Foundation-7
MDA-MB-231	MD Anderson Metastatic Breast 231
ML	Machine Learning
MLP	Multi-Layer Perceptron
mTOR	The Mammalian Target of Rapamycin
MTT	3-(4,5-dimethylthiazol-2-yl)-2,5-diphenyltetrazolium bromide
NF-κB	Nuclear Factor kappa B
NLP	Natural Language Processing
NMF-DR	Non-Negative Matrix Factorization-based Drug Repurposing
PD-L1	Programmed Death-Ligand 1
PDPK1	Phosphoinositide-Dependent Kinase-1
PRs	Progesterone Receptors
QSAR	Quantitative Structure–Activity Relationship
ROC	Receiver Operating Characteristic
RWD	Real World Data
SERD	Selective Estrogen Receptor Degrader
SERM	Selective Estrogen Receptor Modulator
STITCH	Search Tool for Interactions of Chemicals
SUM149	Stephen University Mammalian cell line #149
TCGA	The Cancer Genome Atlas
TNBC	Triple-Negative Breast Cancer
